# The effect of fascial block on opioid consumption in patients with multiple rib fractures: a retrospective observational study

**DOI:** 10.1007/s11739-025-04144-6

**Published:** 2025-10-26

**Authors:** Sossio Serra, Domenico Pietro Santonastaso, Giuseppe Romano, Alessandro Riccardi, Francesca Ortu, Vanni Agnoletti, Mario Guarino, Claudia Sara Cimmino, Michele Domenico Spampinato, Michela Cascio, Raffella Francesconi, Fabio De Iaco

**Affiliations:** 1 UOC Pronto Soccorso E Medicina d’Urgenza, Ospedale Ramazzini, 41012 Carpi, Modena Italy; 2 Anestesia and Intensive Care Unit, Emergency Department, Maurizio Bufalini Hospital, 47521 Cesena, Italy; 3 Emergency Department, Maurizio Bufalini Hospital, 47521 Cesena, Italy; 4 SC Pronto Soccorso E Medicina d’Urgenza, Ospedale Santa Corona, Pietra Ligure, 17027 Savona, Italy; 5 UO Pronto Soccorso E Medicina d’Urgenza, “Ospedale Degli Infermi”, 48018 Faenza, Italy; 6 UOC MEU Ospedale CTO-AORN Dei Colli Napoli, 80131 Naples, Italy; 7 Department of Translational Medicine and for Romagna, University of Ferrara, Via A. Moro 8, 44124 Cona, Ferrara (FE) Italy; 8 Struttura Complessa Di Medicina Di Emergenza, Urgenza Ospedale Maria Vittoria, ASL Città Di Torino, 10144 Turin, Italy

**Keywords:** Pain management, Fascial block, Emergency medicine, Multiple rib fractures, Chest trauma, Opioid consumption

## Abstract

Pain is a common feature in multiple rib fractures. Opioids are the mainstay of analgesic therapy due to their high efficacy, however, can lead to a reduction in respiratory rate and tidal volume, sedation and respiratory failure. Fascial blocks can provide safe, adequate analgesia with only local effects. This study aims to investigate the effect of fascial block on the reduction of total opioid consumption. A retrospective observational study, conducted in the emergency medicine unit (EMU), including all adult, opioid-naïve patients admitted between January 2022 and August 2024 with post-traumatic blunt chest trauma. 103 patients, 26 (27.9%) women, with a median age of 62 years (IQR 56–76), a median Thoracic Trauma Severity Score (TTSS) of 8 (IQR 7–9), a median pain score of 8 on the 0–10 Numeric ranking scale, and 66 patients (60.2%) underwent fascial block. Median total opioid consumption was 92 morphine milligram equivalents (IQR 61.5–140), significantly lower in patients undergoing fascial block (71 (IQR 60–101) vs. 150 (110–210), p < 0.001), despite higher pain on admission (8 (IQR 7–9) vs. 6 (IQR 5–7), p < 0.01), with better pain control and no differences in total length of stay, TTSS or number of rib fractures. This study shows that fascial blocks in patients admitted to the EMU for post-traumatic pain due to multiple rib fractures are associated with a significant reduction in overall opioid consumption and no specific complication, supporting the use of a multimodal approach for better pain control.

## Introduction

Pain is "an unpleasant sensory and emotional experience associated with actual or potential tissue damage”, and a highly subjective phenomenon influenced by biological, psychological, and social factors [1]. Pain treatment is a fundamental human right, and inappropriate pain management leads to unnecessary and harmful suffering [2]. Activation of the sympathetic nervous system due to severe pain leads to tachycardia and hypertension, which increases the risk of ischaemic events and heart failure, especially in patients with pre-existing heart disease. Persistent, untreated pain triggers anxiety and depression [3], leads to reduced mobility with secondary muscle tissue loss and a higher risk of carer dependency, hospitalisation and higher healthcare costs [4]. Pain significantly impairs chest excursion and secretion clearing in rib fractures, favouring pulmonary atelectasis and superimposed bacterial infections, especially in older patients with more rib fractures [5, 6], with an incidence of 6–17% in patients aged < 65 years and 34% in older patients [7, 8]. Adequate analgesia allows deep breathing, effective coughing and chest physiotherapy, reducing atelectasis, pneumonia, and respiratory failure [6]. Opioids are the cornerstone of pain therapy and, due to their high efficacy, are included in the World Health Organisation (WHO) Model List of Essential Medicines [9]. However, high opioid doses can lead to conscious and respiratory depression, hypotension, constipation, nausea and vomiting, urinary retention, tolerance and withdrawal syndrome [10], especially in opioid-naïve, predisposed patients.

Although some authors suggest reserving regional anaesthetic techniques only in patients with inadequate pain control [6], early multimodal analgesic therapy including locoregional anaesthesia may ensure the best outcome, especially in multiple rib fractures with secondary respiratory failure [11–14]. Fascial blocks can provide analgesia avoiding the systemic effects of other drugs [15, 16] with greater efficacy than conventional pharmacological approaches [17]. Despite increasing evidence on the efficacy and safety of fascial blocks, regional anaesthesia is underused, and only 3% of patients with thoracic trauma who may deserve locoregional therapy benefit from this treatment [18]. Although fascial blocks have been studied in surgical settings, there is limited data on the impact of a single fascia block performed in the emergency medicine unit (EMU) on pain perception, opioid consumption and respiratory function throughout the hospital stay [19, 20].

This study aims to evaluate the efficacy of a single fascia block on admission to reduce pain and overall hospital opioid consumption in patients admitted to the emergency medicine unit with only post-traumatic chest pain.

## Methods

This is a retrospective, observational study conducted from January 2022 to August 2024 in the emergency department (ED) emergency medicine unit (EM-U) of a level I trauma centre.

Inclusion criteria were: (1) age over 18 years; (2) closed chest trauma with multiple rib fractures (> 2 fractures) unilateral; (3) severe pain reported as at least six on the numerical rating scale (NRS) of 0–10; (4) spontaneous breathing requiring oxygen therapy; (5) no haemodynamic instability; (6) Glasgow Coma Scale (GCS) = 15; (7) fascial block performed within 24 h of admission. Exclusion criteria were: (1) presence of concomitant traumatic injuries to the limbs, spine, pelvis and/or skull; (2) presence of drained pneumothorax; (3) history of peripheral neuropathy; (4) chronic use of opioids; (5) allergy to amide-type local anaesthetics; (6) severe bilateral rib fractures; (7) severe liver failure; (8) injection site infection.

The fascial blocks performed were the Serratus Anterior Plane Block (SAPB) [14, 15] and the Erector Spinae Plane Block (ESPB) [21], depending on the localisation of the rib fractures (anterolateral or posterior) and the skills of the treating EM physician. Blocks were performed at the bedside with a sterile, ultrasound-guided technique using a portable ultrasound system (SonoSite M-Turbo; SonoSite Inc., WA, USA) with a linear high-frequency probe (6–13 MHz). The injection was performed with an 80 mm long 22-gauge needle (Echoplex + , Vygon, Ecouen-France) and 70–100 mg ropivacaine (1–1.5 mg/kg ideal patient weight) diluted in a total volume of 30 ml with 0.9% saline. In SAPB, the ropivacaine solution was injected between the serratus anterior muscle and the ribs, between the fourth and sixth intercostal spaces. In ESPB, the solution was injected between the erector spinae muscle and the thoracic transverse processes, between T4 and T6.

Clinical data, including age, gender and comorbidities according to the Charlson Comorbidity Index (CCI) [22] were assessed on admission to the EM-U. Trauma severity was assessed using the Thoracic Trauma Severity Score [23]. The vital signs recorded were respiratory rate (RR), peripheral oxygen saturation (SpO2), SpO2/FiO2 ratio [24], systolic blood pressure (SBP), diastolic blood pressure (DBP), and heart rate (HR) at admission and four-times/each day until discharge. Pain was evaluated via an 11-point, 0–10 numerical rating scale (NRS) at admission and four times/each day until discharge, at rest. The treating physicians determined analgesic therapy according to the World Health Organisation analgesic scale for nociceptive pain: mild pain (level 1), non-opioids (paracetamol, non-steroidal anti-inflammatory drugs [NSAIDs]) ± adjuvants; moderate pain (level 2), weak opioids (tramadol) ± non-opioids ± adjuvants; and severe pain (level 3), strong opioids (morphine, fentanyl) ± non-opioids ± adjuvants [25]. For severe pain, patients were treated with a fixed dose of paracetamol and NSAIDs, unless there was a contraindication. Opioids were administered at a fixed dose (transdermal fentanyl, 25 mcg/hour) for severe pain on admission and as intravenous fentanyl or morphine if requested by the patient. Opioid consumption was determined daily in morphine milligram equivalents (MME) using an opioid conversion table [26].

Continuous data were expressed as median and interquartile range; differences between two independent groups were compared using the Mann–Whitney U test, while the Wilcoxon test was used to compare two paired groups. Categorical data were expressed as absolute numbers and percentages: two independent groups were compared using the Pearson chi-square test, while two paired groups were compared using the McNemar test. Statistical analyses were performed with JAMOVI 2.6.24 (The jamovi project (2024).

A formal sample size was calculated. Considering a type I error equal to 0.05, a type II error equal to 0.2, a clinical significative difference between mean equal to 50 MME (considering a standard in-hospital stay of 5 days), a standard deviation in each group equal to 70 and a ratio of sample size between groups 1 and 2 equal to 3, resulting in 84 patients to be included [27].

This study was conducted following the current Declaration of Helsinki and approved by the local ethics committee, the "CET-CEROM—Comitato etico della Romagna”, on 13 December 2023, approval code: 7379/2023. The STROBE checklist was used to prepare this manuscript [28].

## Results

**Fig. 1:**
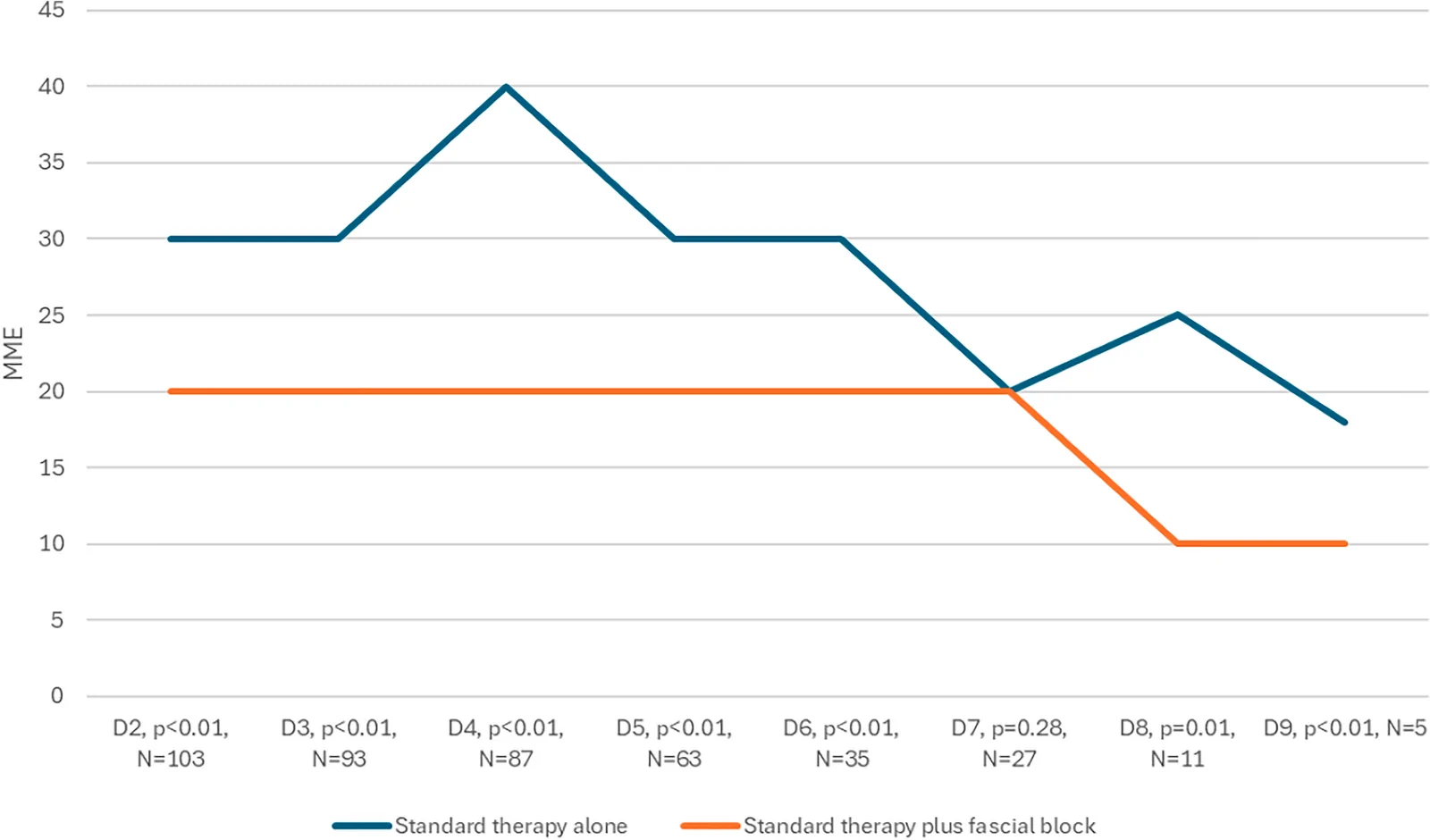
Median opioid consumption in MME during the in-hospital stay according to the analgesic strategy adopted (standard therapy alone, blue line, vs. standard therapy plus fascial block, orange line). On the vertical axis, the median MME administered, on the horizontal axis, days from admission, p for the comparison between the two doses of opioids, total number of patients evaluated (example: D2, p < 0.01, N = 103: Day 2, MME significantly different, 103 patients included)

Throughout the study period, 103 patients fulfilled the inclusion and exclusion criteria and were included in the study. The median age of patients was 62 years (IQR 56–76), with 75 men (73%) and a median CCI of 2 (IQR 1–4). Patients showed a median of 1 fractured right rib (IQR 0–5) and three fractured left ribs (IQR 0–6), and a median TTSS of 8 (IQR 7–9). On admission, patients had a median SpO2/FiO2 ratio of 424 (IQR 310–457) and a median pain score of 8 (IQR 6–8). The median length of stay was 4 days (IQR 3–5) and the median total opioid consumption was 92 MME (IQR 61.5–140). A fascial block was performed in 66 patients (64%), 19 ESPB (28.8%) and 47 SAPB (71.2%), with a median dose of 80 mg ropivacaine (IQR 80–100). No patient died in hospital or had to be admitted to the intensive care unit (ICU) after admission to the emergency department. 1 patient developed delirium, three patients developed constipation, two patients developed somnolence with a Richmond Agitation Sedation Scale (RASS) of -2 due to opioid ingestion without the need for airway support, two patients developed pneumonia requiring oxygen therapy with a high flow nasal cannula. No complications related to fascial blockade have been reported. See Table 1 for the general characteristics of the included patients. Compared to standard therapy alone, patients undergoing fascial blockade had a higher NRS (8, IQR 7–9, vs. 6, IQR 5–7, p < 0.001) at admission, reported a lower NRS during the first 2 days, required a lower total dose of opioids (71 MME, IQR 60–101.8 vs. 150 MME, IQR 110–210), with no differences in age, CCI, TTSS, SaO2/FiO2 at any time point and LOS. No differences in pain relief or MME were observed between ESPB and SAPB. See Table 2 for general characteristics of included patients after standard therapy alone vs. standard therapy plus fascial block, Table 3 for NRS and Fig. 1 for opioid requirements during hospitalisation.

**Table 1:** General characteristics of included patients

Characteristic	N = 103
Age, years	62 (56, 76)
Sex, male	75 (73%)
Mild cognitive impairment	5 (4.9%)
COPD	10 (9.8%)
CCI	2 (1–2)
Right ribs fractured	0 (0, 5)
Left ribs fractured	3 (0, 6)
Sternal fracture	7 (6.8%)
TTSS	8 (7, 9)
Type of fascial block	
ESPB	19 (18%)
SAPB	47 (46%)
Ropivacaine dose, mg	80 (80–100)
NRS at admission	8 (6–8)
Total opioid consumption, MME	92 (62, 140)
Complicances	
Delirium	1 (1.0%)
Stipsi	3 (2,9)
Pneumonia	2 (1.9%)
Stupor	2 (1.9%)
LOS, days	4 (3–5)

All data are expressed in median (IQR) or number (percentage)

*CCI* Charlson Comorbidity Index, *COPD* Chronic Obstructive Pulmonary Disease, *ESPB* Erector Spinae Plane Block, *LOS* length of Stay, *MME* morphine milligram equivalent, *NRS* Numeric Ranking Scale, *TTSS* Thoracic Trauma Severity Score, *SAPB* Serratus Anterior Plane Block

**Table 2:** General characteristics of patients included according to the analgesic therapy, standard therapy or standard therapy plus fascial block

Results	Fascial block performed		
Characteristic	No, N = 37 (36%)	Yes, N = 66 (64%)	p-value
Sex, male	26 (70%)	49 (74%)	0.66
Mild cognitive impairment	3 (8.1%)	2 (3.0%)	0.25
COPD	7 (19%)	3 (4.6%)	0.03
CCI	2 (1–4.3)	2 (1–4)	0.43
Right ribs fractured	0 (0–5)	0 (0–5)	0.82
Left ribs fractured	3 (0–6)	4 (0–7)	0.41
Sternal fracture	2 (5.4%)	5 (7.6%)	0.67
TTSS	8 (7–8)	8 (6–9)	0.79
NRS at admission	6 (5–7)	8 (7–9)	< 0.01
Total opioid consumption, MME	150 (110–210)	71 (60–101)	< 0.01
Complicance			0.3
Delirium	1 (2.7%)	0 (0%)	
Stipsi	2 (5,4)	2 (3%)	
Pneumonia	1 (2.7%)	1 (1.5%)	
Stupor	2 (5.4%)	0 (0%)	
LOS, days	3 (3–4)	4 (3–5)	0.08

All data are expressed in median (IQR) or number (percentage)

*CCI* Charlson Comorbidity Index, *COPD* Chronic Obstructive Pulmonary Disease, *ESPB* Erector Spinae Plane Block, *LOS* Length of Stay, *MME* morphine milligram equivalent, *NRS* Numeric Ranking Scale, *TTSS* Thoracic Trauma Severity Score, *SAPB* Serratus Anterior Plane Block

**Table 3:** Pain level declared at each time point after enrolment

	Fascial block performed		
	No, N = 37 (36%)	Yes, N = 66 (64%)	p-value
NRS at admission	*5.0* **6.0** *7.0*	*7.0* **8.0** *9.0*	< 0.01
NRS 6 h	3.0 **4.0** 5.0	2.0 **3.0** *4.0*	0.02
NRS 12 h	0.7 **3.0** 5.0	2.0 **3.0** *4.0*	1.05
NRS 18 h	2.0 **3.0** 4.1	0.0 **2.0** *3.0*	< 0.01
NRS 24 h	2.0 **3.0** 4.0	0.0 **3.0** *3.0*	0.02
NRS 1d2	2.0 **4.0** 6.0	0.0 **1.0** *3.0*	< 0.01
NRS 2d2	2.0 **3.0** 4.0	0.0 **1.0** *3.0*	< 0.01
NRS 3d2	2.0 **3.0** 4.0	0.0 **1.0** *3.0*	< 0.01
NRS 4d2	0.0 **3.0** 4.0	0.0 **1.0** *3.0*	0.04
NRS 1d3	0.0 **2.0** 3.0	0.0 **1.5** *3.0*	0.28
NRS 2d3	0.0 **2.0** 3.3	*0.0* **1.0** *3.0*	0.28
NRS 3d3	0.0 **1.0** 2.0	*0.0* **2.0** *3.0*	0.42
NRS 4d3	0.0 **1.5** 3.0	*0.0* **2.0** *3.0*	0.71
NRS 1d4	2.0 **4.0** 6.0	*0.0* **1.0** *3.0*	< 0.01
NRS 2d4	2.0 **4.0** 4.0	*0.0* **2.0** *3.0*	< 0.01
NRS 3d4	1.7 **3.0** 4.0	*0.0* **2.0** *3.0*	0.02
NRS 4d4	0.0 **3.0** 4.0	0.0 **2.0** 3.0	0.08
NRS 1d5	0.0 **2.0** 3.0	0.0 **0.0** 3.0	0.22
NRS 2d5	0.0 **2.0** 3.3	0.0 **0.0** 3.0	0.12
NRS 3d5	0.0 **1.0** 2.0	0.0 **0.0** 3.0	0.78
NRS 4d5	0.0 **1.5** 3.0	0.0 **3.0** 3.0	0.91
NRS 1d6	0.0 **1.0** 3.0	0.0 **3.0** 3.2	0.48
NRS 2d6	0.0 **3.0** 3.0	1.2 **3.0** 4.2	0.36
NRS 3d6	0.0 **1.0** 3.0	1.2 **3.5** 4.6	0.15
NRS 4d6	0.0 **0.0** 3.0	0.5 **4.0** 4.8	0.13
NRS 1d7	0.0 **0.0** 3.0	0.0 **1.0** 3.0	< 0.01
NRS 2d7	0.0 **1.0** 5.2	0.0 **3.0** 3.0	1.00
NRS 3d7	0.0 **2.0** 3.2	3.0 **3.0** 3.0	0.34
NRS 4d7	0.0 **2.5** 3.1	0.0 **1.5** 3.0	0.75
NRS 1d8	1.0 **2.0** 2.2	1.0 **2.5** 4.0	0.88
NRS 2d8	1.8 **3.0** 3.0	0.0 **2.5** 5.0	1.00
NRS 3d8	2.0 **2.5** 3.6	0.0 **2.5** 5.0	1.00
NRS 4d8	2.0 **3.0** 4.0		
NRS 1d9	3.0 **3.0** 3.6	1.0 **1.0** 1.0	0.11
NRS 2d9	2.2 **3.0** 3.8	3.0 **3.0** 3.0	1.00
NRS 3d9		0.0 0.0 0.0	
NRS 4d9			

For each day (d) from 1 to 9 (d1 to d9), 4 measurements per day were collected 1 to 4, and expressed in numeric rating severity between 0 to 10. NRS are expressed in median (central, bold number) and interquartile range

## Discussion

In patients with rib fractures, pain management is a crucial aspect of care, as adequate analgesia improves respiratory function, can reduce the risk of complications, and promote overall recovery [5, 12]. However, the most recent Eastern Association for the Surgery of Trauma (EAST) guidelines on non-surgical treatment and analgesia in patients with multiple rib fractures do not recommend in favour of or against locoregional analgesia due to paucity and quality of evidence [12, 29].

### Opioid consumption

Our study has shown that fascial blocks were associated with lower overall opioid consumption during hospitalisation, which is crucial for minimising the risk of opioid-related side effects such as nausea, vomiting and respiratory depression. These results are consistent with the systematic review and meta-analysis published in 2024 by Xiao et al., [30], the RCT by Teksen et al. in 2021 [31], and the SABRE RCT, 2024, [32] showing that peripheral nerve block and in particular SAPB allows better pain control at rest and during cough/movement in the first 24 h, with a significantly lower need for rescue analgesics and opioids [30–32]. SAPB appears to be as effective as more invasive pain management techniques such as thoracic epidural analgesia (TEA) in reducing opioid consumption in the first 48 h, with no differences in patient preparation and positioning time, time to perform the block or patient satisfaction. However, patients receiving TEA were more often treated with diazepam [33]. Similarly, ESPB was found to be effective in pain management with the same opioid consumption and lower incidence of hypotension than TEA, further supporting the benefit of fascial blocks in reducing opioid consumption without compromising pain relief [34, 35].

In the RCT by Ramesh et al. (2024), patients receiving ESPB required fewer rescue analgesics compared to the sham group, suggesting that the ESPB not only provided better pain control but also reduced the need for additional opioid administration [36].

Several authors reported the effectiveness of ESPB in reducing the administration of opioids in the emergency department [20], not only for rib and spinal fractures [14, 37, 38] but also for mechanical pain [39], burns [40], herpes zoster [41], renal colic [42] and acute pancreatitis [43]. However, in most studies fascial blocks were performed by anaesthetists in different settings (intensive care unit or operating theatre) [31, 33–35], and only a few are high quality RCTs in which fascial blocks were performed by emergency physicians [32, 36]. In addition, studies reported on the effects of fascial block in the first 24–48 h, while the present study is the first examining total opioid consumption during the entire hospital stay in the emergency medicine unit (EM-U), providing new insights into the efficacy of these analgesic techniques.

### Pain management

Our results are consistent with those of previous studies showing that both ESPB and SAPB are associated with better pain control at rest and during cough or movement within the first 24 h compared with standard therapy alone, with no significant differences in pain scores 24 h after fascial block execution (see Table 3) [30, 31, 36].

No differences in early pain relief when comparing SAPB and TEA [34, 44] or ESPB and thoracic paravertebral block (TPVB) [45] or TEA [46] are demonstrated. Despite Lunden et al. (2023) found a greater efficacy of TEA compared to SAPB at rest and during movement at 0.5, 1, 2 and 12 h after block, no differences were noted at other time points [33], and fascial blocks are emerging as potential effective alternatives for pain management, although evidence on other outcomes is limited [19].

Although the present study was not designed to study the differences in terms of pain relief between ESPB and SAPB, no differences were found at any time point between the two blocks. In an RCT by El Malla et al., 2022, both ESPB and SAPB patients experienced a significant reduction in pain scores, with higher efficacy of ESPB in terms of pain intensity both at rest and when coughing, and higher diaphragmatic excursion 2, 6, 12 and 24 h after the block was performed [47]. In an RCT conducted in patients undergoing thoracic surgery (mainly video-assisted thoracoscopy, thoracotomy and mastectomy), no differences were noted between SAPB and ESPB in effective pain management, opioid consumption, and need for rescue analgesics [48, 49], despite ESPB showed a higher quality of recovery and a longer time to first opioid requirement [50].

### Respiratory function and Length of Stay

In the present study, no differences were found in SpO2/FiO2 ratio at baseline or during hospitalisation, length of stay or overall complications between standard therapy only and standard therapy plus fascial block. However, literature shows contrasting data on the effect of fascial block on lung ventilation. The meta-analysis by Xiao et al. [30] found no effect of fascial block on PaO2 and forced vital capacity (FVC). Few of the included studies reported the effects of fascial block on respiratory rate (RR), PaCO2, peak expiratory flow rate (PEFR) or PaO2/FiO2. SAPB showed a positive impact on SpO2 24 h after the block [32] and on incentive spirometry volume (ISV) 3 h after the block [51]. ESPB has been shown to increase ISV in the first 24 h after the procedure, with the improvement lasting up to 72 h [38]. Compared to TEA, SAPB showed a smaller positive effect on forced expiratory volume in 1 s in per cent (FEV1%) at 0.5, 1, 2 and 12 h after the procedure, with no differences at 24 and 48 h [34] and no differences in the increase in inspiratory volume [45]. Compared to TPVB, ESPB showed no differences in PaO2/FiO2 ratio, PcCO2, RR, forced vital capacity (FVC) or FEV1% [35, 47] and no differences in mean SpO2 at 30 min and up to 24 h [46]. Compared to SAPB, ESPB showed a higher diaphragmatic excursion from 30 min to 24 h after the block, although no further data on respiratory function are available [48].

At the same time, no differences were reported in the literature regarding complications such as delirium and pneumonia [33], and length of hospital and intensive care stay between standard therapy and fascial block [31, 33, 38], although other authors reported a positive effect of ESPB on length of stay [19].

## Limitations and future research

Despite our study offering significant insights into the analgesic efficacy of ESPB and SAPB, it has some limitations that must be addressed. Despite patients included being higher than the calculated sample size and this manuscript being among the larger retrospective studies, the efficacy of fascial blocks may be under- or over-estimated by the present study. Although no significant differences were noted at baseline between groups, patients were not randomly assigned to treatment and fascial blocks were executed based on the technical proficiency of the treating EM physician. The type of fascial block may not have aligned with the anatomical location of the fractured ribs (antero-lateral versus posterior), resulting in a potential lack of analgesic effect. A future RCT or an observational study with an established protocol of fascial block according to the localisation of rib fractures may elicit a greater efficacy of this technique in MME consumption reduction. Only patients with a single fascial block were included, and repeated fascial blocks or catheter-based local anaesthetic infusion may have a different impact on opioid consumption and pain scores. Patients with severe bilateral rib fractures were excluded to avoid introducing biases in our study. In the present study, we evaluated the efficacy of ropivacaine at doses of up to 1.5 mg/kg in a volume of 40 ml. Bilateral fascial blocks may need lower ropivacaine dosages per side, potentially resulting in a reduced analgesic effect due to the lower concentration and/or lower total volume of the solution administered. A further study comparing the effect of bilateral fascial blocks with different dosages may help to elucidate the efficacy of this technique on pain management and opioid consumption. We reported only pain during rest, however, data regarding effective pain management during deep inspiration, respiratory physiotherapy, coughing, or physical activity could elucidate the effects of fascial block on pain and other outcomes. We have documented data regarding the SpO2/FiO2 ratio, and the impacts of fascial blocks on respiratory rate, inspiratory volume, end-tidal CO2, arterial CO2, forced vital capacity, and FEV1% remain unexamined. None of the studies, including the current one, gathered patient-reported outcome measures (PROMs) such as discomfort from fascial blocks, and the comparison of conventional analgesia versus fascial block on quality of life during hospitalisation, and patient satisfaction.

## Conclusion

In conclusion, early multimodal analgesic strategies, including fascial blocks, in the pain management of patients admitted to the emergency medicine unit for multiple rib fractures appear to be effective in reducing overall opioid consumption during hospitalisation, with higher analgesic efficacy and no specific side effects.

## Data Availability

Data are available under appropriate request.

## References

[1] Raja SN, Carr DB, Cohen M, Finnerup NB, Flor H, Gibson S, Keefe FJ, Mogil JS, Ringkamp M, Sluka KA, Song XJ, Stevens B, Sullivan MD, Tutelman PR, Ushida T, Vader K (2020) The revised international association for the study of pain definition of pain: concepts, challenges, and compromises. Pain 161(9):1976–1982. 10.1097/j.pain.0000000000001939 32694387 PMC7680716

[2] https://www.apsoc.org.au/PDF/Publications/DeclarationOfMontreal_IASP.pdf

[3] Michaelides A, Zis P (2019) Depression, anxiety and acute pain: links and management challenges. Postgrad Med 131(7):438–444. 10.1080/00325481.2019.1663705 31482756

[4] Sinatra R (2010) Causes and consequences of inadequate management of acute pain. Pain Med (Malden, Mass) 11(12):1859–1871. 10.1111/j.1526-4637.2010.00983.x 21040438

[5] Flagel BT, Luchette FA, Reed RL, Esposito TJ, Davis KA, Santaniello JM, Gamelli RL (2005) Half-a-dozen ribs: the breakpoint for mortality. Surgery 138(4):717–725. 10.1016/j.surg.2005.07.022 16269301

[6] May L, Hillermann C, Patil S (2016) Rib fracture management. BJA Educ 16(1):26–32. 10.1093/bjaceaccp/mkv011

[7] Bergeron E, Lavoie A, Clas D, Moore L, Ratte S, Tetreault S, Lemaire J, Martin M (2003) Elderly trauma patients with rib fractures are at greater risk of death and pneumonia. J Trauma 54(3):478–485. 10.1097/01.TA.0000037095.83469.4C 12634526

[8] Dehghan N, de Mestral C, McKee MD, Schemitsch EH, Nathens A (2014) Flail chest injuries: a review of outcomes and treatment practices from the National Trauma Data Bank. J Trauma Acute Care Surg 76(2):462–468. 10.1097/TA.0000000000000086 24458051

[9] www.who.int/groups/expert-committee-on-selection-and-use-of-essential-medicines/essential-medicines-lists

[10] Pandharipande P (2025) Pain control in the critically ill adult patient. In: UpToDate, Connor RF (Ed), Wolters Kluwer. Accessed 16 Feb 2025

[11] De Simone B, Chouillard E, Podda M, Pararas N, de Carvalho Duarte G, Fugazzola P, Birindelli A, Coccolini F, Polistena A, Sibilla MG, Kruger V, Fraga GP, Montori G, Russo E, Pintar T, Ansaloni L, Avenia N, Di Saverio S, Leppäniemi A, Lauretta A, Catena F (2024) The 2023 WSES guidelines on the management of trauma in elderly and frail patients. World J Emerg Surg: WJES 19(1):18. 10.1186/s13017-024-00537-8 38816766 PMC11140935

[12] Galvagno SM, Smith CE, Varon AJ, Hasenboehler EA, Sultan S, Shaefer G, To KB, Fox AD, Alley DE, Ditillo M, Joseph BA, Robinson BR, Haut ER (2016) Pain management for blunt thoracic trauma: a joint practice management guideline from the Eastern Association for the Surgery of Trauma and Trauma Anesthesiology Society. J Trauma Acute Care Surg 81(5):936–951. 10.1097/TA.0000000000001209 27533913

[13] De Iaco F, Gandolfo E, Guarino M, Riccardi A, Serra S (2022) Terapia del dolore in urgenza e sedazione procedurale: Manuale SAU. [Self-published]. ISBN 979-8819663554

[14] Serra S, Santonastaso DP, Romano G, Riccardi A, Nigra SG, Russo E, Angelini M, Agnoletti V, Guarino M, Cimmino CS, Spampinato MD, Francesconi R, Iaco F (2024) Efficacy and safety of the serratus anterior plane block (SAP block) for pain management in patients with multiple rib fractures in the emergency department: a retrospective study. Eur J Trauma Emerg Surg Off Publ Eur Trauma Soc 50(6):3177–3188. 10.1007/s00068-024-02597-6 PMC11666617 39020130

[15] Southgate SJ, River GF, Herbst MK (2024) Ultrasound-guided serratus anterior blocks. StatPearls Publishing, In StatPearls

[16] Thiruvenkatarajan V, Cruz Eng H, Adhikary SD (2018) An update on regional analgesia for rib fractures. Curr Opin Anaesthesiol 31(5):601–607. 10.1097/ACO.0000000000000637 30020155

[17] Ho AM, Karmakar MK, Critchley LA (2011) Acute pain management of patients with multiple fractured ribs: a focus on regional techniques. Curr Opin Crit Care 17(4):323–327. 10.1097/MCC.0b013e328348bf6f 21716105

[18] Cheema FA, Chao E, Buchsbaum J, Giarra K, Parsikia A, Stone ME, Kaban JM (2019) State of rib fracture care: a NTDB review of analgesic management and surgical stabilization. Am Surg 85(5):474–478 31126359

[19] Gamberini L, Moro F, Dallari C, Tartaglione M, Mazzoli CA, Allegri D, Scquizzato T, Chiarini V, Coniglio C, Brogi E (2025) Regional anesthesia modalities in blunt thoracic trauma: a systematic review and Bayesian network meta-analysis. Am J Emerg Med 89:199–208. 10.1016/j.ajem.2024.12.029 39740311

[20] Abdelhamid K, ElHawary H, Turner JP (2020) The use of the Erector Spinae Plane block to decrease pain and opioid consumption in the emergency department: a literature review. J Emerg Med 58(4):603–609. 10.1016/j.jemermed.2020.02.022 32245689

[21] Siddiqui N, Krishnan S, Dua A, Cascella M (2025) Erector Spinae Plane Block. StatPearls Publishing, In StatPearls 31424889

[22] Charlson ME, Pompei P, Ales KL, MacKenzie CR (1987) A new method of classifying prognostic comorbidity in longitudinal studies: development and validation. J Chronic Dis 40(5):373–383. 10.1016/0021-9681(87)90171-8 3558716

[23] Daurat A, Millet I, Roustan JP, Maury C, Taourel P, Jaber S, Capdevila X, Charbit J (2016) Thoracic trauma severity score on admission allows to determine the risk of delayed ARDS in trauma patients with pulmonary contusion. Injury 47(1):147–153. 10.1016/j.injury.2015.08.031 26358517

[24] Bonaventura A, Mumoli N, Mazzone A, Colombo A, Evangelista I, Cerutti S, Brivio L, Vecchié A, Bonomi A, Para O, Bellizzi A, Cei M, Fenu P, Tangianu F, Dentali F, Cei F (2022) Correlation of SpO2/FiO2 and PaO2/FiO2 in patients with symptomatic COVID-19: an observational, retrospective study. Intern Emerg Med 17(6):1769–1775. 10.1007/s11739-022-02981-3 35460432 PMC9034256

[25] Anekar AA, Hendrix JM, Cascella M (2023) WHO Analgesic Ladder. StatPearls Publishing, In StatPearls 32119322

[26] De Iaco F, Mannaioni G, Serra S, Finco G, Sartori S, Gandolfo E, Sansone P, Marinangeli F (2022) Equianalgesia, opioid switch and opioid association in different clinical settings: a narrative review. Eur Rev Med Pharmacol Sci 26(6):2000–2017. 10.26355/eurrev_202203_28349 35363351

[27] MedCalc Software Ltd (2025) Sample size for comparison of means (Version 23.3.7) [Computer software]. https://www.medcalc.org/calc/sample-size-comparison-of-means.php. Accessed 1 Sept 2025

[28] von Elm E, Altman DG, Egger M, Pocock SJ, Gøtzsche PC, Vandenbroucke JP, STROBE Initiative (2008) The strengthening the reporting of observational studies in epidemiology (STROBE) statement: guidelines for reporting observational studies. J Clin Epidemiol 61(4):344–349. 10.1016/j.jclinepi.2007.11.008 18313558

[29] Mukherjee K, Schubl SD, Tominaga G, Cantrell S, Kim B, Haines KL, Kaups KL, Barraco R, Staudenmayer K, Knowlton LM, Shiroff AM, Bauman ZM, Brooks SE, Kaafarani H, Crandall M, Nirula R, Agarwal SK Jr, Como JJ, Haut ER, Kasotakis G (2023) Non-surgical management and analgesia strategies for older adults with multiple rib fractures: A systematic review, meta-analysis, and joint practice management guideline from the Eastern Association for the Surgery of Trauma and the Chest Wall Injury Society. J Trauma Acute Care Surg 94(3):398–407. 10.1097/TA.0000000000003830 36730672

[30] Xiao DL, Xi JW (2023) Efficacy of peripheral nerve blocks for pain management in patients with rib fractures: a systematic review and meta-analysis. Eur Rev Med Pharmacol Sci 27(3):899–910. 10.26355/eurrev_202302_31183 36808336

[31] Tekşen Ş, Öksüz G, Öksüz H, Sayan M, Arslan M, Urfalıoğlu A, Gişi G, Bilal B (2021) Analgesic efficacy of the serratus anterior plane block in rib fractures pain: a randomized controlled trial. Am J Emerg Med 41:16–20. 10.1016/j.ajem.2020.12.041 33383266

[32] Partyka C, Asha S, Berry M, Ferguson I, Burns B, Tsacalos K, Gaetani D, Oliver M, Luscombe G, Delaney A, Curtis K (2024) Serratus anterior plane blocks for early rib fracture pain management: the SABRE randomized clinical trial. JAMA Surg 159(7):810–817. 10.1001/jamasurg.2024.0969 38691350 PMC11063926

[33] Lundén AR, Tarkkila P (2024) An ultrasound-guided serratus anterior plane block with continuous local anaesthetic infusion and epidural analgesia for rib fracture pain. Acta Anaesthesiol Scand 68(3):394–401. 10.1111/aas.14355 37934716

[34] Mostafa SF, Eid GM (2023) Ultrasound guided erector spinae plane block versus thoracic epidural analgesia in traumatic flail chest, a prospective randomized trial. J Anaesthesiol Clin Pharmacol 39(2):250–257. 10.4103/joacp.joacp_416_21 37564847 PMC10410029

[35] Singh S, Avinash R, Jaiswal S, Kumari A (2023) Comparison of safety and efficacy of thoracic epidural block and erector spinae plane block for analgesia in patients with multiple rib fractures: a pilot single-blinded, randomised controlled trial. Indian J Anaesth 67(7):614–619. 10.4103/ija.ija_844_21 37601936 PMC10436724

[36] Ramesh S, Ayyan SM, Rath DP, Sadanandan DM (2024) Efficacy and safety of ultrasound-guided erector spinae plane block compared to sham procedure in adult patients with rib fractures presenting to the emergency department: a randomized controlled trial. Acad Emerg Med 31(4):316–325. 10.1111/acem.14820 37843475

[37] Adhikary SD, Liu WM, Fuller E, Cruz-Eng H, Chin KJ (2019) The effect of erector spinae plane block on respiratory and analgesic outcomes in multiple rib fractures: a retrospective cohort study. Anaesthesia 74(5):585–593. 10.1111/anae.14579 30740657

[38] Ahiskalioglu A, Kocak AO, Doymus O, Sengun E, Celik M, Alici HA (2018) Erector spinae plane block for bilateral lumbar transverse process fracture in emergency department: a new indication. Am J Emerg Med 36(10):1927.e3-1927.e4. 10.1016/j.ajem.2018.06.072 29980485

[39] Apibunyopas Y, Ho B (2019) Ultrasound-guided erector spinae plane (ESP) block: a novel intervention for mechanical back pain in the emergency department. CJEM 21(2):302–305. 10.1017/cem.2018.469 35118628

[40] Ueshima H, Otake H (2018) Continuous erector spinae plane block for pain management of an extensive burn. Am J Emerg Med 36(11):2130.e1-2130.e2. 10.1016/j.ajem.2018.07.014 30072048

[41] Tekin E, Ahiskalioglu A, Aydin ME, Sengun E, Bayramoglu A, Alici HA (2019) High-thoracic ultrasound-guided erector spinae plane block for acute herpes zoster pain management in emergency department. Am J Emerg Med 37(2):375.e1-375.e3. 10.1016/j.ajem.2018.10.028 30340986

[42] Aydin ME, Ahiskalioglu A, Tekin E, Ozkaya F, Ahiskalioglu EO, Bayramoglu A (2019) Relief of refractory renal colic in emergency department: a novel indication for ultrasound guided erector spinae plane block. Am J Emerg Med 37(4):794.e1-794.e3. 10.1016/j.ajem.2018.12.042 30595427

[43] Elkoundi A, Eloukkal Z, Bensghir M, Belyamani L, Lalaoui SJ (2019) Erector spinae plane block for hyperalgesic acute pancreatitis. Pain medicine (Malden, Mass) 20(5):1055–1056. 10.1093/pm/pny232 30476275

[44] Beard L, Hillermann C, Beard E, Millerchip S, Sachdeva R, Gao Smith F, Veenith T (2020) Multicenter longitudinal cross-sectional study comparing effectiveness of serratus anterior plane, paravertebral and thoracic epidural for the analgesia of multiple rib fractures. Reg Anesth Pain Med 45(5):351–356. 10.1136/rapm-2019-101119 32165553 PMC8408582

[45] Elawamy A, Morsy MR, Ahmed MAY (2022) Comparison of thoracic erector spinae plane block with thoracic paravertebral block for pain management in patients with unilateral multiple fractured ribs. Pain Physician 25(6):483–490 36122257

[46] El-Sherbiny SM, Kamal RA, Elhadary IH, Abdallah MYY (2022) Erector spinae plane block versus thoracic epidural block as analgesic techniques for chest trauma: a randomised controlled trial. Res Opinion Anesthesia Intensive Care 9(3):220–227. 10.4103/roaic.roaic_64_21

[47] El Malla DA, Helal RAEF, Zidan TAM, El Mourad MB (2022) The effect of erector spinae block versus serratus plane block on pain scores and diaphragmatic excursion in multiple rib fractures. A prospective randomized trial. Pain Med (Malden, Mass) 23(3):448–455. 10.1093/pm/pnab214 34240173

[48] Wu W, Xu H, Chen X, He W, Shi H (2023) Comparison of erector spinae plane block and serratus anterior plane block for postoperative analgesia in uniportal thoracoscopic lobectomy: a randomized controlled trial. BMC Anesthesiol 23(1):394. 10.1186/s12871-023-02353-0 38041029 PMC10691001

[49] Ekinci M, Ciftci B, Gölboyu BE, Demiraran Y, Bayrak Y, Tulgar S (2020) A randomized trial to compare Serratus Anterior Plane Block and Erector Spinae Plane Block for pain management following thoracoscopic surgery. Pain Med (Malden, Mass) 21(6):1248–1254. 10.1093/pm/pnaa101 32417925

[50] Finnerty DT, McMahon A, McNamara JR, Hartigan SD, Griffin M, Buggy DJ (2020) Comparing erector spinae plane block with serratus anterior plane block for minimally invasive thoracic surgery: a randomised clinical trial. Br J Anaesth 125(5):802–810. 10.1016/j.bja.2020.06.020 32660716

[51] Sadauskas V, Fofana M, Brunson D, Choi J, Spain D, Quinn JV, Duanmu Y (2024) Serratus anterior plane block improves pain and incentive spirometry volumes in trauma patients with multiple rib fractures: a prospective cohort study. Trauma Surg Acute Care Open 9(1):e001183. 10.1136/tsaco-2023-001183 38881827 PMC11177771

